# Arthroscopic treatment for femoroacetabular impingement yields favourable patient‐reported outcomes and method survivorship at 10‐year follow‐up

**DOI:** 10.1002/ksa.12511

**Published:** 2024-10-22

**Authors:** Sarantos Nikou, Joel Sturesson, Ida Lindman, Louise Karlsson, Axel Öhlin, Eric Hamrin Senorski, Mikael Sansone

**Affiliations:** ^1^ Department of Orthopaedics Institute of Clinical Sciences, Sahlgrenska Academy, University of Gothenburg Gothenburg Sweden; ^2^ Department of Orthopaedic Surgery South Älvsborg Hospital Borås Sweden; ^3^ Research, Education, Development & Innovation, Primary Health Care Vastra Gotaland Sweden; ^4^ Department of Health and Rehabilitation Institute of Neuroscience and Physiology, Sahlgrenska Academy, University of Gothenburg Gothenburg Sweden

**Keywords:** FAIS, femoroacetabular impingement syndrome, hip, hip arthroscopy, patient‐reported outcome measures

## Abstract

**Purpose:**

To compare the outcomes of hip arthroscopy for femoroacetabular impingement syndrome (FAIS) preoperatively and at minimum 10‐year follow‐up using patient‐reported outcome measures (PROMs).

**Methods:**

A total of 128 patients with FAIS were prospectively included. The patients underwent arthroscopic surgery for FAIS between 2011 and 2013 and had a minimum of 10‐year follow‐up. The International Hip Outcome Tool short version (iHOT‐12) was the primary outcome. Secondary outcomes were the Copenhagen Hip and Groin Outcome Score (HAGOS), the European Quality of Life‐5 Dimensions Questionnaire (EQ‐5D), the European Quality visual analogue scale (EQ VAS), the Hip Sports Activity Scale (HSAS) for physical activity level, the Visual Analogue Scale (VAS) for overall hip function and a single question regarding overall satisfaction with the surgery. The Wilcoxon signed rank test was used to compare pre‐ and postoperative PROMs.

**Results:**

There was a significant improvement (*p* < 0.001) of iHOT‐12, HAGOS subscales, EQ‐5D, EQ VAS and VAS for overall hip function. A total of 83% of the patients were satisfied with their surgery. The survivorship of hip arthroscopy, defined as nonconversion to total hip arthroplasty (THA), at the end of the follow‐up period was 77%.

**Conclusion:**

Patients undergoing arthroscopic treatment for FAIS reported statistically significant and clinically relevant improved outcomes at 10‐year follow‐up.

**Level of Evidence:**

Level IV, case series.

**Abbreviations**
EQ VAS: European Quality visual analogue scale
EQ‐5D: European Quality of Life‐5 Dimensions Questionnaire
FAIS: femoroacetabular impingement syndrome
HAGOS: Copenhagen Hip and Groin Outcome Score
HSAS: Hip Sports Activity Scale
iHOT‐12: International Hip Outcome Tool
mHHS: modified Harris Hip Score
MIC: minimal important change
NSAID: nonsteroid anti‐inflammatory drugs
PASS: patient acceptable symptomatic state
PROMs: patient‐reported outcome measures
RCTs: randomized controlled studies
SD: standard deviation
THA: total hip arthroplasty
VAS: Visual Analogue Scale

## INTRODUCTION

Femoroacetabular impingement syndrome (FAIS) is a common cause of hip and groin pain among young and physically active individuals [9]. In patients with cam morphology, the labral and cartilage injury caused by shearing forces can lead to osteoarthritis [12, 30].

Treatment of FAIS can be either nonsurgical with supervised personalized physiotherapy, patient education and activity modifications or surgical with the use of hip arthroscopy together with rehabilitation [3, 6]. Randomized controlled studies (RCTs) have reported superior outcomes of hip arthroscopy compared with physiotherapy; however, the majority have a short follow‐up period of 1 year [1, 14, 19, 28]. The arthroscopic treatment for FAIS is a well‐established treatment option [15]. The aim of the surgery is to restore the hip joint anatomy by treating the bony irregularities (cam/pincer) and addressing any soft tissue damage that is associated with this condition [26].

The long‐term outcomes of hip arthroscopic treatment for FAIS have not been extensively reported. Previous studies have evaluated predictive factors of hip arthroscopy results for FAIS, concluding that high grade of cartilage injury, older age and longer symptom duration are associated with worse outcomes [7, 8, 38]. Several studies have reported good 2‐ and 5‐year outcomes of hip arthroscopic treatment for FAIS [17, 27, 28, 33]. However, there are only a few studies evaluating the long‐term outcomes of hip arthroscopy using modern validated patient‐reported outcome measures (PROMs) [23, 24, 33, 43].

The purpose of this study was to report the 10‐year patient‐reported hip function outcomes of hip arthroscopic treatment for FAIS using PROMs. The secondary aim was to investigate the survivorship of hip arthroscopy defined as nonconversion to total hip arthroplasty (THA) at a 10‐year follow‐up. The hypothesis was that at a 10‐year follow‐up, a statistically and clinically meaningful improvement in PROMs would be observed.

## METHODS

**Figure 1:**
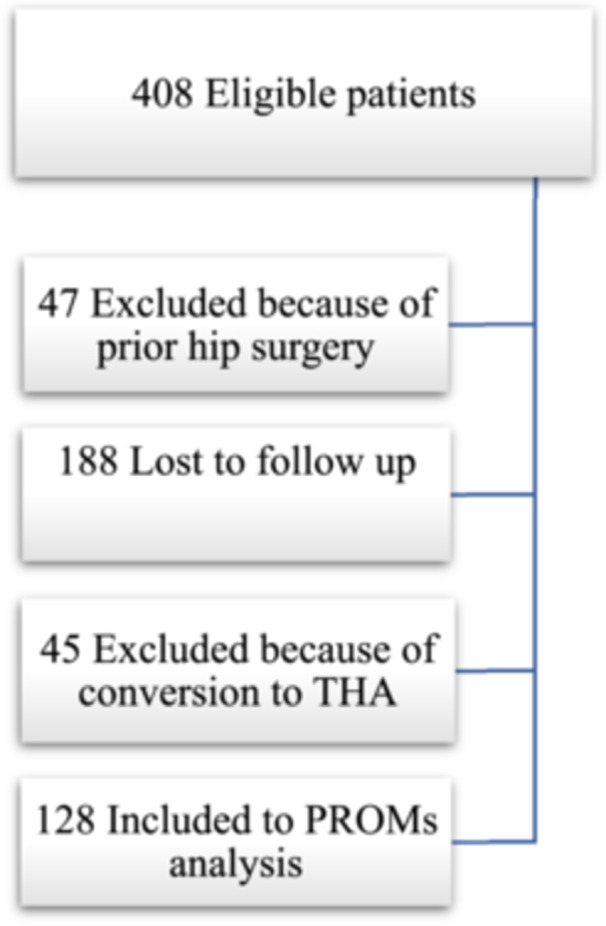
Flowchart of included and excluded patients.

Patients with FAIS who underwent hip arthroscopy between January 2011 and December 2013 were prospectively registered in a local hip arthroscopy registry. Surgeries were performed by three experienced surgeons at two hospitals in Gothenburg, Sweden. Patients were eligible for inclusion if they had a diagnosis of FAIS and had failed nonoperative treatment before proceeding to surgery. Exclusion criteria for PROMs analysis were previous hip surgery and withdrawn consent as well as conversion to THA. A total of 128 patients (151 hips) were included in the final PROMs analysis (Figure 1).

The demographic and perioperative information, including age, sex, uni‐ or bilateral surgery, operated side, surgical and traction time and Konan classification grade of acetabular chondral damage, was reported by the treating surgeon. Information regarding reoperations and conversion to THA was retrieved from patient journals.

Descriptive statistics were used to present demographic data. The data were reported as mean, median, standard deviation (SD) and range for ordinal data. For nominal data, relative and absolute frequencies were used. Nonparametric statistical testing was used to compare paired means for continuous PROM data not normally distributed. To compare PROMs between preoperative and 2‐year follow‐up, the Wilcoxon signed rank test was used. The level of significance was set at *p *˂ 0.05. Considering that 10 points of score change in International Hip Outcome Tool (iHOT‐12) are clinically relevant, an SD of 21 points (as Jonasson et al. [22] has previously suggested) and an *α*‐value of 0.05, the sample size calculation revealed that a power of >90% would be reached with 75 patients [31, 33, 41]. The survivorship rate was calculated by dividing the number of hips not receiving THA within 10 years by the number of hips at risk of receiving THA. The Statistical Package for the Social Sciences (IBM SPSS statistics, version 28.0.1.1) was used to statistically analyse the patient data and PROMs.

The patients completed the following PROMs preoperatively and at the 10‐year follow‐up:

- International Hip Outcome Tool short version (iHOT‐12), a shorter version of the iHOT‐33, which measures both health‐related quality of life and changes after treatment in young, active patients with hip disorders [16].
- Copenhagen Hip and Groin Outcome Score (HAGOS). The HAGOS questionnaire consists of six subscales: Symptoms, Pain, Function in daily living (ADL), Function in sport and recreation (Sport/Rec), Participation in Physical Activities (PA) and hip and/or groin‐related Quality of Life (QOL). The scores of each subscale are summed up and transformed to a 0–100 scale. A score of 0 represents severe hip and/or groin problems, while a score of 100 represents no hip and/or groin problems. With an interclass correlation coefficient (ICC) between 0.81 and 0.89 for the six subscales, the test–retest reliability of the Swedish version of HAGOS is found to be very good [39].
- European Quality of Life–5 Dimensions Questionnaire (EQ‐5D) and European Quality of Life–Visual Analogue Scale (EQ VAS), a standardized instrument evaluating health‐related quality of life [35]. This PROM is a descriptive system that comprises five dimensions: mobility, self‐care, usual activities, pain/discomfort and anxiety/depression. Each dimension has five levels: no problems, slight problems, moderate problems, severe problems and extreme problems. Previous studies have shown that this instrument is valid and reliable [18].
- Hip Sports Activity Scale (HSAS), a PROM measuring the level of physical activity in patients with FAIS. The HSAS scale consists of nine different levels, from lowest being ‘No recreational or competitive sports’ to highest ‘Competitive sports (elite level)' [29]. The Swedish version of HSAS has been found to be reliable (test‐retest reliability, ICC 0.930) and to have good content validity when compared to the Tegner score (*r* = 0.794) [34].
- VAS scale for hip function.
- A question regarding satisfaction with surgery (yes/no).

Patients exceeding the minimal important change (MIC) were reported, with the use of a distribution‐based technique, setting the cut‐off value at 0.5 times the SD of the score change [31, 40]. The number of patients achieving the acceptable symptomatic state (PASS) for the six HAGOS subscales and the iHOT‐12 was reported. The PASS is defined as the highest level of symptom beyond which patients consider themselves well [21]. In previous studies, the PASS values are estimated and set for the iHOT‐12 63, HAGOS pain 68.8, HAGOS symptoms 62.5, HAGOS‐ADL 82.5, HAGOS‐PA 43.8, HAGOS‐Sport/Rec 60.9 and HAGOS‐QoL 42.5 [21, 22, 32].

Informed consent was obtained from each patient in the study. Ethical approval for the study was granted by the Regional Ethical Review Board in Gothenburg (registration number EPN 2019‐06050).

## SURGICAL TECHNIQUE

The hip arthroscopy procedures were performed in a supine position using one anterolateral and one midanterior portal. Axial traction was used to gain access to the central compartment. The peripheral compartment was approached through a capsulotomy made longitudinal to the capsule fibres and the iliofemoral ligament. This approach may potentially decrease the risk of iatrogenic‐induced hip laxity postoperatively.

To assess the correct reshaping of the femoral head‐neck junction cam morphologies were resected under the guidance of intraoperative fluoroscopy. An ‘over‐the‐top’ technique was used for the correction of pincer morphology [20]. Labral tears were treated with either debridement or suture, depending on the type and size of the tear. Depending on the location and morphology, the cartilage lesions were treated with either debridement or microfracture. No capsular closures were performed. Postoperatively full weight‐bearing adjusted in relation to pain was allowed immediately, but patients were recommended crutches for up to 4 weeks when walking outdoors. All patients were assigned to supervised rehabilitation. The patients were furthermore prescribed diclofenac 50 mg orally three times a day for three weeks postoperatively to prevent heterotopic ossification.

## RESULTS

There were 128 patients (151 hips) included in the final PROMs analysis. The follow‐up rate, defined as the percentage of eligible patients who were not lost to follow‐up was 48% (173/361). The method survivorship rate was 77%. Demographic data are presented in Table 1.

**Table 1:** Patient demographics and perioperative data.

Total number of patients	128
Total number of hips	151
Operated side, right/left/bilateral (%)	81/64/6 (53/42/4)
Age—mean (SD)	37 years (13.0)
Male/female (%)	74/54 (57.8/42.2)
Follow‐up time (SD)	138 (4) months
Symptom duration—median (min‐max)	24 (2–204) months
Operation time—mean (SD)	74 (17) minutes
Traction time—mean (SD)	9 (7) minutes

Of the included 151 hips, 42 had isolated cam morphology (28%). Two hips had isolated pincer morphology (1%) and 79 hips had both cam and pincer morphology (52%). The labrum was sutured in 16 hips (Table 2). Ten patients had exposed bare bone in the acetabulum corresponding to Konan type 4 (Table 3).

**Table 2:** Arthroscopic procedures performed.

Procedure	Yes (%) of the total number of hips
Cam	42 (27.8)
Pincer	2 (1.3)
Cam + pincer (Combined)	79 (52.3)
Labral suture	16 (10.6)
Microfracture	7 (4.6)
Labral resection	9 (6)
Teres ligament resection	1 (0.7)

*Note*: Twenty‐eight hips (18.5%) were not specified by the operating surgeon.

**Table 3:** Incidence of cartilage damage, classification according to Konan et al.

Cartilage damage classification by Konan et al.	Hips (% of visualized hips)
0	31 (22.5)
1a	9 (6.0)
1b	1 (0.7)
2	27 (17.9)
3a	14 (9.3)
3b	2 (1.3)
4a	5 (3.3)
4b	3 (2.0)
4c	2 (1.3)
Not visualized	30 (19.9)

*Note*: Twenty‐seven hips were not specified by the operating surgeon.

When comparing the preoperative PROMs results to those obtained at 10‐year follow‐up a statistically significant increase in all outcome measures except for HSAS was found. At the 10‐year follow‐up, 104 patients (83%) reported that they were satisfied with the surgery (Table 4).

**Table 4:** Outcome data.

PROMs	Preoperative, Mean (SD)	10 year follow‐up, mean (SD)	Change mean (SD)	Preoperative, median	10 years, median	Change median (range)	% patients MIC	% Exceeding PASS	*p* Value
iHOT‐12	44.1 (18.4)	72.3 (29.6)	28.2 (19.9)	44.5	85.8	34.8 (−42 to 90.3)	76	70	<0.001
HAGOS‐symptoms	52.0 (18.4)	76.0 (21.8)	23.9 (16.9)	53.6	82.1	25 (−53.6 to 67.9)	77	77	<0.001
HAGOS‐pain	57.0 (19.8)	83.0 (22,1)	25.9 (18,3)	57.5	90	27.5 (−37.5 to 82.5)	77	80	<0.001
HAGOS‐ daily activity	61.60 (24.2)	81.1 (27.4)	19.5 (13.8)	60	93.8	16.3 (−66.3 to 100)	66	84	<0.001
HAGOS sport	41.7 (21.5)	75.1 (28.4)	33.4 (23.7)	40.6	85.9	37.5 (−53.1 to 90.6)	77	74	<0.001
HAGOS‐ physical activity	31.9 (28.6)	74.5 (31.5)	42.6 (30.1)	25	87.5	50 (−75 to 100)	75	80	<0.001
HAGOS‐ quality of life	31.8 (18.2)	72.1 (28.0)	40.3 (28.5)	35	80	45.0 (−35 to 95)	79	84	<0.001
EQ‐5D‐TTO	0.8005 (0.1350)	0.9051 (0.1070)	0.1046 (0.07400)	0.8545	0.9349	0.0721 (−0.9349 to 0.5434)	NA	NA	<0.001
EQ VAS	68.0 (18.6)	78.0 (17.0)	10.0 (7.1)	70	80	10.0 (−95 to 70)	NA	NA	<0.001
HSAS	3.1 (2.0)	3.4 (1.9)	0.4 (0.3)	3	3	1 (−5 to 6)	NA	NA	n.s
VAS‐ overall hip function	49.0 (20.5)	74.2 (24.4)	25.1 (17.8)	50	85	25 (−60 to 85)	NA	NA	<0.001
Satisfied with surgery %		83							

Abbreviations: EQ‐5D, European Quality of Life‐5 Dimensions Questionnaire; HAGOS, Copenhagen Hip and Groin Outcome Score; HSAS, Hip Sports Activity Scale; iHOT‐12, International Hip Outcome Tool; MIC, minimal important change; PASS, patient acceptable symptomatic state; VAS, visual analogue scale.

The median preoperative iHOT‐12 score was 44.5 compared with 85.8 at 10‐year follow‐up. The calculated MIC value for iHOT‐12 was 10.0. Of all patients, 76% (97/128) exceeded MIC, while 69% (88/128) exceeded the PASS level of 63.0 postoperatively.

A statistically significant improvement (*p* < 0.001) of all HAGOS subscales was observed. For HAGOS subscales, the calculated MIC values were 8.5 for symptoms, 9.2 for pain, 6.9 for ADL, 11.8 for Sports/Rec, 15.0 for PA and 14.2 for QoL (Table 4).

No significant change in HSAS levels was observed at the 10‐year follow‐up, compared with baseline. The median HSAS levels preoperatively and at 10‐year follow‐up was 3.1 versus 3.4.

## DISCUSSION

The most important finding of this study was the statistically significant improvement of PROMs at 10‐year follow‐up after hip arthroscopic treatment for FAIS compared with preoperative values. Clinically meaningful results were achieved as demonstrated by the number of patients exceeding the MIC and PASS values for iHOT‐12 and HAGOS scores. The survivorship of the method was durable (77%) at the end of the follow‐up period.

Previous studies have reported similar PROMs at medium‐ and long‐term follow‐up [24, 28]. In a study with 177 athletes treated with hip arthroscopy for FAIS, the included patients were followed for a mean period of 127 months. The patients demonstrated a significant improvement in PROMs at a minimum 10‐year follow‐up with an 85.7% survivorship of the method, defined as no conversion to THA [10]. Another study, including 393 hips followed patients treated for FAIS for a mean period of 7.5 years [13]. The hip joint preservation rate was 90.4% (SD ± 1.7%; 95% confidence interval [CI]: 87.1–93.7), with a durable improvement of PROMs at the final follow‐up. Interestingly the authors performed a subgroup analysis investigating the surgical results of three distinct groups treated with different techniques. Both the survivorship rate and PROMs were better for the group treated with modern arthroscopic techniques compared with open or semi‐open techniques. That is in concordance with the results of the present study, where all included patients were treated with arthroscopic techniques.

Carton et al. [5] reported on a group of 119 patients with FAIS treated with arthroscopic osteochondroplasty with a minimum of 10‐year follow‐up. Statistically significant improvements were seen in modified Harris Hip Score (mHHS), 36‐Item Short Form Health Survey (SF‐36) and Western Ontario and McMaster Universities Osteoarthritis Index (WOMAC) scores, with high satisfaction rate (90%) observed at 10 years after surgery. The hip arthroscopy survivorship rate for this group was 91.4%, and the satisfaction rate was 90%. Tönnis grade 1 was associated with a lower survivorship rate of 80%, and the authors could not find any association between the increased risk of THA conversion and the age and sex of the patients. However, the PROMs used in that study do not satisfactorily reflect the level of function and activity for the young and active population.

In this study, all PROMs except for HSAS were improved at 10‐year follow‐up compared with baseline. Similar PROMs were reported by Kaldau et al. [23] for a group of 84 patients with FAIS treated with hip arthroscopy with a median follow‐up time of 82.9 months. At the end of the follow‐up period, 18% of the patients had undergone THA, which is in concordance with the present study. In the present study, there was a statistically significant improvement of EQ‐5D and HAGOS values while HSAS was unchanged. This can possibly be explained by the fact that in the long term, many of these patients do not continue at the same level of physical activity they had before the FAIS symptoms presented because of their increased age. However, the HAGOS subscales for sport and physical activity exhibited a significant improvement compared with baseline (sport 41.7 vs. 75.1, physical activity 31.9 vs. 74.5), thus making it difficult to draw any substantial conclusions.

The 2‐ and 5‐year follow‐ups for the same group of patients included in this study have previously been reported [33, 37]. The PROMs did not decline over time and the patient satisfaction rate at 10‐year follow‐up is at the same level compared with 5 years after surgery (83% vs. 84.6%). Survivorship at the 10‐year follow‐up had marginally declined compared to the previous follow‐up.

The results in this study are comparable to other studies in which capsular repair and labral suturing are more commonly used [2, 11]. Although the study is not designed to evaluate the effect of these procedures, the present study shows that good results can be achieved with different techniques [4].

The strengths of this study are the long follow‐up period, the large number of patients as well as the use of PROMs suitable for a young, active population. There are, however, several limitations to this study. There is no control group, thus not allowing a direct comparison to the PROMs of the treatment group included in this study. Hence, there is a risk of confounding factors affecting the reported PROMs. However, this is a case series study with prospective enrolment of patients where the whole group of patients treated has been eligible for inclusion.

Another limitation of this study is the follow‐up rate of 48%, which could be attributed to the long follow‐up period and could affect the internal validity of the study. However, the relatively low response rate (RR) is not uncommon in registry studies [42]. In a scoping review of RRs in clinical quality registries, the authors found that the average RR in Scandinavian registry studies at 10‐year follow‐up was about 51% (±10.7) [36]. A previous study compared patient reported outcomes between responders and initial nonresponders in the hip arthroscopy registry also used for the purpose of this study; the authors concluded that there was no difference between the two groups except for the patient satisfaction rate [25]. Hence, the results reported seem to be in concordance with previous studies.

Moreover, there is no radiographic 10‐year follow‐up of the patients included in this study. That depends on the fact that patient data used for this study were extracted from a local registry that does not include a radiographic examination. However, it would be interesting to investigate if the patients who are less satisfied at the final follow‐up have more radiographic signs of cartilage injury than the patients who are satisfied.

## CONCLUSION

Patients undergoing arthroscopic treatment for FAIS reported statistically significant and clinically relevant improved outcomes at 10‐year follow‐up.

## AUTHOR CONTRIBUTIONS

All authors contributed to the study conception and design. Material preparation, data collection and analysis were performed by Sarantos Nikou and Joel Sturesson. The first draft of the manuscript was written by Sarantos Nikou and all authors commented on previous versions of the manuscript. All authors read and approved the final manuscript.

## ETHICS STATEMENT

The study was approved by the Swedish Ethical Review Authority. All the procedures being performed were part of the routine care. Informed consent was obtained from all individual participants included in the study.

## CONFLICT OF INTEREST STATEMENT

The authors declare no conflict of interest.

## Data Availability

Derived data supporting the findings of this study are available from the corresponding author on request.
